# Dual Effect of Low-Molecular-Weight Bioregulators of Bacterial Origin in Experimental Model of Asthma

**DOI:** 10.3390/life12020192

**Published:** 2022-01-27

**Authors:** Svetlana V. Guryanova, Olga B. Gigani, Georgii O. Gudima, Anastasiya M. Kataeva, Natalya V. Kolesnikova

**Affiliations:** 1 Shemyakin-Ovchinnikov Institute of Bioorganic Chemistry of Russian Academy of Sciences, Ministry of Science and Higher Education of the Russian Federation, 117997 Moscow, Russia; 2 Medical Institute, Peoples’ Friendship University of Russia (RUDN University), Ministry of Science and Higher Education of the Russian Federation, 117198 Moscow, Russia; giolga@yandex.ru (O.B.G.); akata1991@mail.ru (A.M.K.); 3 National Research Center-Institute of Immunology of the Federal Medico-Biological Agency, 115522 Moscow, Russia; g.gudima@nrcii.ru; 4 Department of Clinical Immunology, Kuban State Medical University, Ministry of Health of the Russian Federation, 350063 Krasnodar, Russia; nvk24071954@mail.ru

**Keywords:** asthma, experimental asthma models, innate immunity, TLR4, NOD2, muramyl peptide, glucosaminylmuramyldipeptide, GMDP, lipopolysaccharide, LPS

## Abstract

Asthma is one of the most common noncommunicable diseases, affecting over 200 million people. A large number of drugs control asthma attacks, but there is no effective therapy. Identification of reasons for asthma and preventing this disease is a relevant task. The influence of bacterial components is necessary for the normal development of the immune system and the formation of an adequate immune response to antigens. In the absence of microorganisms or their insufficient exposure, the prerequisites are formed for excessive reactivity to harmless antigens. In the present study, we analyzed cellular and humoral factors in a standard mouse model of OVA-induced asthma modified by 5-fold intraperitoneal injection of bacterial cell wall fragments of glucosaminylmuramyl dipeptide (GMDP) 5 μg/animal or 1 μg lipopolysaccharide (LPS) per animal for 5 days before sensitization by ovalbumin (OVA). Preliminary administration of LPS or GMDP to animals significantly reduced goblet cells as well as the number of neutrophils, lymphocytes, and eosinophils in bronchoalveolar lavage, wherein GMDP corrected neutrophilia to a 2-fold degree, and LPS reduced the severity of eosinophilia by 1.9 times. With OVA administration of GMDP or LPS at the sensitization stage, an increase in the total number of bronchoalveolar lavage cells due to neutrophils, macrophages, lymphocytes, and eosinophils in relation to the group with asthma without GMDP or LPS was observed. The administration of GMDP or LPS to normal mice without asthma for 5 days had no statistically significant effect on the change in the number and population composition of cells in bronchoalveolar lavage in comparison with the control group receiving PBS. As a result of a study in a mouse model of asthma, a dual effect of LPS and GMDP was established: the introduction of LPS or GMDP before sensitization reduces neutrophilia and eosinophilia, while the introduction of LPS or GMDP together with an allergen significantly increases neutrophilia and eosinophilia. The study of the immunoglobulin status shows that in normal-asthma mice, GMDP and LPS slightly increase IgA in bronchoalveolar lavage; at the same time, in the asthma model, injections of GMDP or LPS before sensitization contribute to a significant decrease in IgA (2.6 times and 2.1 times, respectively) in BALF and IgE (2.2 times and 2.0 times, respectively) in blood serum. In an experimental model of asthma, the effect of GMDP and LPS was multidirectional: when they are repeatedly administered before sensitization, the bacterial components significantly reduce the severity of the allergic process, while in the case of a joint injection with an allergen, they increase the influx of macrophages, lymphocytes, and neutrophils into the lungs, which can aggravate the course of pathological process. Thus, the insufficient effect of antigens of a bacterial nature, in particular, with prolonged use of antibiotics can be compensated for by substances based on low-molecular-weight bioregulators of bacterial origin to establish the missing signals for innate immunity receptors, whose constant activation at a certain level is necessary to maintain homeostasis.

## 1. Introduction

Asthma is one of the main noncommunicable diseases that affects both children and adults: in 2019, the number of asthma patients was 262 million, and there were 461,000 deaths from the disease [1]. More than 40 million new cases are registered annually, while a statistically significant inverse correlation was found between the socio-demographic index and the incidence of asthma [2].

Recent advances in next-generation sequencing technology have revealed the key role of the lung and gut microbiota in the pathogenesis of asthma and other diseases of the respiratory tract [3]. Bacterial communities characteristic of patients with asthma have been identified; the bacteria *Fusobacterium, Lachnospira, Veillonella,* and *Rothia* are more common in patients with bronchial asthma than in healthy people [4]. Normally, the microorganism migrates to the lungs through microaspiration, is carried by the cells of the ciliated epithelium, and is removed by phagocytosis by alveolar macrophages, which present processed bacterial antigens to the cells of the immune system and, through cytokines and mediators, maintain the balance and stability of the microecology of the lungs. The microbiome contributes to the formation and maturation of the immune system and the formation of homeostatic relationships, supporting innate and adaptive immunity [5]. In turn, the host’s organism affects microorganisms, regulating the production of immunoglobulin IgA; antimicrobial components, such as defensins; and providing tolerance, thus contributing to the preservation of commensal microflora on the mucosal surface [6,7,8].

In the case of impaired functions of the cells of the pulmonary epithelium, for example, damage to the cilia of the mucous membrane or an excessive amount of secreted mucus, observed during allergic processes, colonization by microorganisms occurs, which can lead to infectious pathologies [9]. Systemic inflammation or inhibition of the phagocytic activity of macrophages can also cause changes in the lung microbiome [3,4,5,8].

When analyzing the microbiota of the upper respiratory tract, it was found that, in normal, healthy people, microbial immigration from the oral cavity is an important source of the microbiome of the lungs and stomach, and the distribution of taxonomic groups in the lungs correlates with microorganisms of the oral cavity and not the nasopharynx [10]. In addition, the diversity of the intestinal microflora at an early stage of life is a prerequisite for the development of the immune system, significantly reducing the frequency of asthma and other allergic diseases [11,12,13,14], and the use of broad-spectrum antibiotics in childhood significantly increases the likelihood of allergic asthma in adulthood [4,15,16,17]. The manifestation of asthma and atopy is also influenced by a genetic predisposition: in the study of single-nucleus polymorphisms (SNPs) of 3062 children with asthma, multiple single SNPs were found, including the TLR and NLR genes, as well as significant interactions between these genes associated with phenotypic manifestations of asthma [18,19]. The revealed polymorphisms of the TLR and NLR genes affect the binding of these receptors to their ligands and to numerous adapter proteins, modifying intracellular signaling pathways involved during asthma, chronic obstructive pulmonary disease, and others [19,20].

With the natural decay of microflora, a large number of compounds are formed, which, interacting with the receptors of innate immunity, trigger innate reactions designed to limit the invasion of the pathogen. The most studied are lipopolysaccharides (LPS)—TLR agonists and muramylpetides (MDP)—NLR ligands.

Lipopolysaccharides (LPS) and muramyl peptides (MDP) are low-molecular-weight components of bacterial cell walls and are actively involved in modulating the host immune response by interacting with innate immunity receptors present on bronchial epithelial cells and immunocompetent cells [21,22,23]. The result of this interaction is the activation of intracellular processes, which consists, on the one hand, in the induction of pro-inflammatory cytokines and mediators and, on the other hand, in the control of the intensity of the inflammatory process and its completion [24,25,26]. In this case, the lipopolysaccharide, being a fragment of gram-negative bacteria, triggers anti-infectious protection through TLR4 and muramyl peptides, which are part of both gram-positive and gram-negative bacteria, through NOD2 receptors. TLR4 are located on the outer membrane of the cell, and NOD2 in the cytoplasm; both types of receptors thus form a multilevel defense against bacteria [27,28]. Dysregulation of these interactions leads to a variety of pathologies, such as chronic inflammation, autoimmune and allergic reactions, and asthma.

Experimental study of the causes of asthma in humans has ethical limitations, in connection with which the task of developing and studying a model of asthma in laboratory animals becomes urgent. The most common rodent model of asthma is induced ovalbumin (OVA) [29,30,31]. In this case, the OVA-induced mouse model mimics the stages of asthma in humans and explains the physiological, cellular, and molecular mechanisms underlying this pathology.

In the present study, based on an OVA-induced mouse model of asthma, we studied the effect of TLR4 and NOD2 receptor agonists, lipopolysaccharide, and glucosaminylmuramyl dipeptide (GMDP) on humoral and cellular factors of allergic inflammation.

## 2. Materials and Methods

### 2.1. Animal Care

Male 4-week-old BALB/C mice (Pushchino, Russia) were kept in a routine room with a 12-h light/dark cycle under defined pathogen-free conditions and had free access to food and water. All experiments were performed in accordance with the Geneva Convention “International Guiding Principles for Biomedical Involving Animals” (Geneva, 1990), as well as the Declaration of Helsinki by the World Medical Association on the humane treatment of animals (2000 revision). The study was approved by the Ethics Committee of the Peoples’ Friendship University of Russia, N11S/20 (Moscow, Russia).

### 2.2. OVA-Induced Allergic Asthma in Mice

Mice were randomly divided into eight groups of 6 animals in each group (Table 1). In the first group of mice, allergic airway inflammation (Asthma) caused by OVA was induced. For this, mice were sensitized on day 0 with an intraperitoneal injection (ip) of 20 μg OVA (Merck, Germany) and 1 mg aluminum hydroxide (Merck, Germany) in 0.2 mL of sterile saline (PBS) on days 0, 14, and 21, and then, 80 μg of OVA in 50 μL of sterile PBS was injected intranasal on days 27, 28, and 35. On day 37, bronchoalveolar lavage, blood, and lungs were collected.

In groups 2, 3, 6, and 7, animals were injected intraperitoneally 5 days before sensitization with 5 μg/animal of GMDP (JSC Peptek, Russia) in PBS or 1 μg/animal of LPS (Ultra-pure, Invivogen) in PBS. In groups 2 and 3, ovalbumin was then administered similarly to group 1 (Asthma). In groups 4 and 5, GMDP and LPS, during sensitization animals were intraperitoneally injected at 5 μg/animal GMDP in PBS or 1 μg/animal LPS in PBS together with 20 μg OVA and 1 mg aluminum hydroxide in 0.2 mL of sterile saline solution. The control group of mice was injected intraperitoneally and intranasal with sterile PBS according to the protocol.

### 2.3. The Number and Analysis of Cells in BAL

Animals were sacrificed 48 h after the last administration of OVA, and samples of bronchoalveolar lavage fluid (BAL) were collected by intratracheal instillation of 700 μL of PBS three times. LBAL was centrifuged to collect whole cells into a pellet with 0.5 mL of PBS, and supernatants were carefully removed and stored at –80 °C for ELISA. The total number of leukocyte cells, neutrophils, lymphocytes, monocytes, and eosinophils in BAL was counted on a hematology counter (Beckman Coulter LH 750, Brea, CA, USA). The analysis of cell populations of dried smears was carried out after staining according to Romanovsky—Giemsa (Gemstandart, St. Petersburg, Russia) using immersion.

### 2.4. Collecting Blood

Blood samples were taken from the heart and were kept at room temperature for 30 min, followed by centrifugation at 1000 g for 15 min, and serum was isolated by aspiration. The separated serum was stored at −80 °C for the later quantitative determination of total IgE and OVA-specific IgG1 and IgG2a.

### 2.5. ELISA

Quantification of IgA in BALF as well as OVA-specific IgG1 and IgG2a and total serum IgE were measured using mouse ELISA kits (ThermoFisher Waltham, MA, USA) according to the manufacturer’s instructions.

### 2.6. Histology

The lungs were fixed in 10% paraformaldehyde solution at 1 h at room temperature, and 4 micron sections were prepared. The samples were visualized using an inverted light microscope (Nikon Eclipse E200, Tokyo, Japan).

### 2.7. Statistical Analysis

Results are presented as mean ± standard error of the mean. Differences were statistically significant using an unpaired two-tailed Student’s *t*-test (for comparison between the two groups). Data analysis was performed using the GraphPad Prism 8.0.2 software package (GraphPad Software, Inc., La Jolla, CA, USA). *p* < 0.05 was considered to indicate a statistically significant difference.

## 3. Results

**Figure 1:**
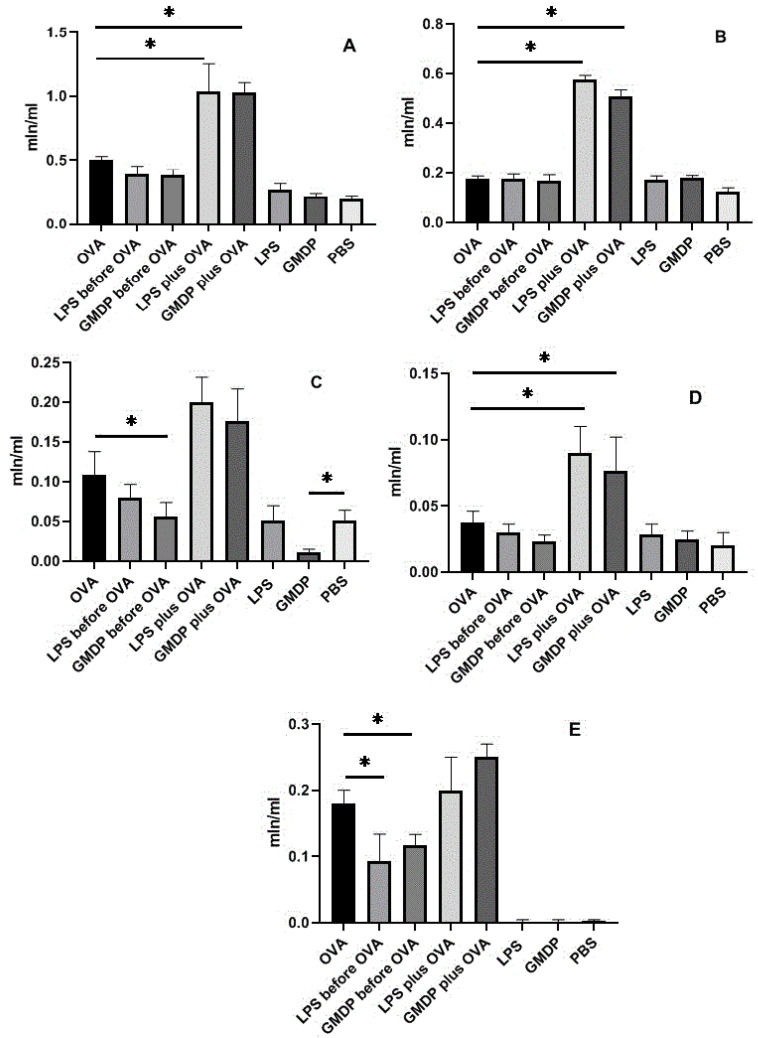
Cell count and analysis in bronchoalveolar lavage fluid. (**A**) The total number of cells in BAL; (**B**) macrophages; (**C**) neutrophils; (**D**) lymphocytes; (**E**) eosinophils; * *p* < 0.05.

**Figure 2:**
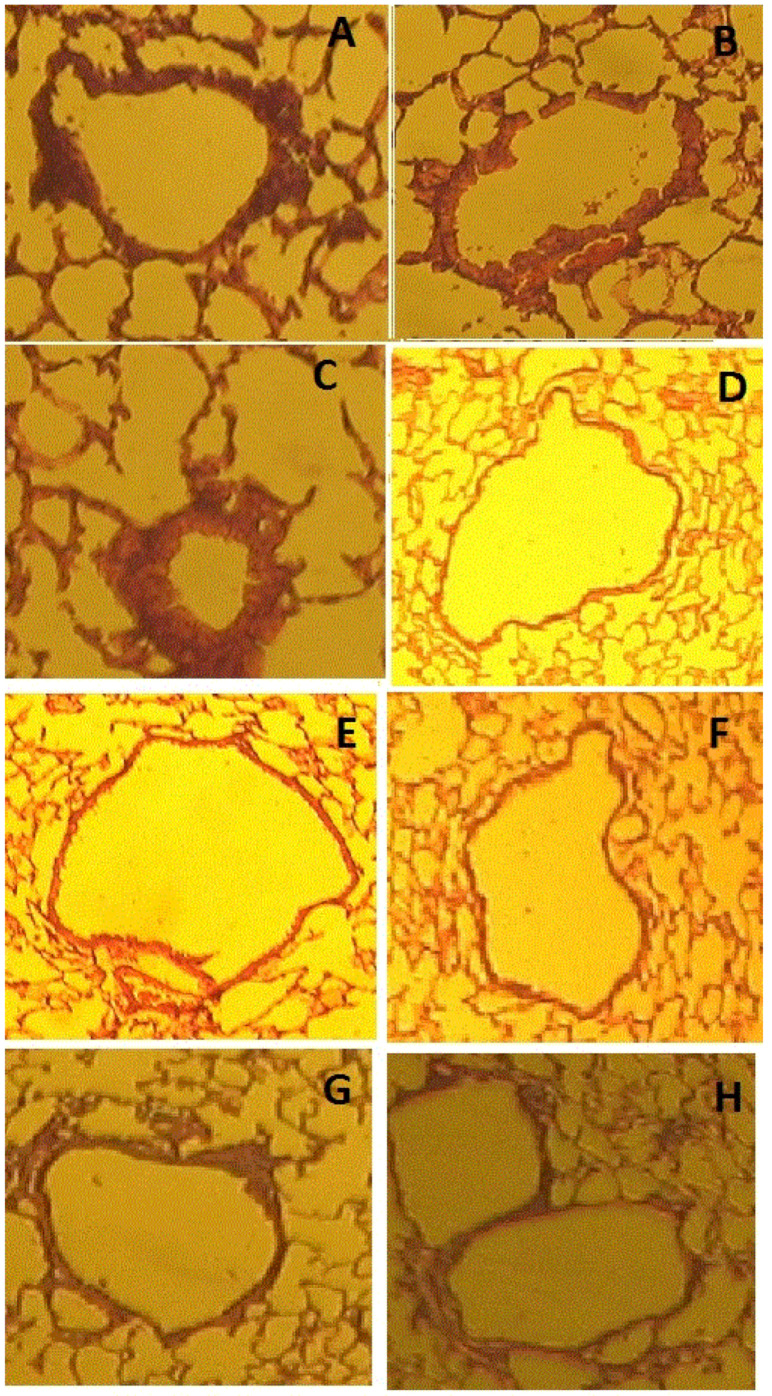
Influence of preliminary intraperitoneal administration of LPS and GMDP on pathological changes in the lungs caused by OVA. (**A**) Asthma (OVA); (**B**) exposure to 1 μg LPS in conjunction with OVA; (**C**) exposure to 5 μg GMDP together with OVA; (**D**) control animals receiving only PBS; (**E**) animals that received LPS at the preliminary stage before OVA sensitization; (**F**) animals that received GMDP at the preliminary stage before OVA sensitization; (**G**) animals that received only LPS; (**H**) animals that received only GMDP. Five times administration of LPS and GMDP prior to OVA sensitization showed a protective effect.

The standard asthma model (group 1, Table 1), modified by five-fold intraperitoneal injection of GMDP or LPS five days before sensitization (groups 2 and 3), significantly decreases eosinophils and neutrophils in bronchoalveolar lavage compared with the classical asthma model (Figure 1) and goblet cells (Figure 2), while the change in the total number of cells in BAL due to an increase in macrophages and lymphocytes is not statistically significant.

The administration of GMDP or LPS (groups 6 and 7) to normal mice without asthma for five days (groups 6 and 7) did not have a statistically significant effect on the change in the number and population composition of cells in BAL compared with the control group (group 8) receiving PBS. Preliminary (before OVA sensitization) administration of LPS or GMDP to animals with asthma significantly reduced the number of neutrophils, lymphocytes, and eosinophils, and GMDP significantly corrected neutrophilia (a 2-fold decrease), and LPS reduced the severity of eosinophilia by 1.9 times. When combined with OVA intraperitoneal injection of GMDP or LPS at the stage of sensitization (groups 4 and 5), an increase in the total number of bronchoalveolar lavage cells occurred due to neutrophils, macrophages, lymphocytes, and eosinophils in relation to the group with asthma (group 1). The results obtained are consistent with the data of studies according to which the intranasal administration of OVA together with muramyldipeptide—the ligand of the NOD2 receptor—increased the number of eosinophils in the mouse lungs, and intranasal and intraperitoneal administration of LPS in a rat model of asthma increased the number of monocytes, neutrophils, and eosinophils in the lungs [32,33]. It can be concluded that, regardless of the route of administration, the combined use of an antigen with LPS or muramylpeptides contributes to an increase in the number of eosinophils in the lungs.

Thus, as a result of a study in a mouse model of asthma, a dual effect of LPS and GMDP was established: multiple administration of LPS and GMDP before sensitization reduces neutrophilia and eosinophilia, while joint administration of LPS or GMDP together with an allergen significantly increases neutrophilia and eosinophilia.

**Figure 3:**
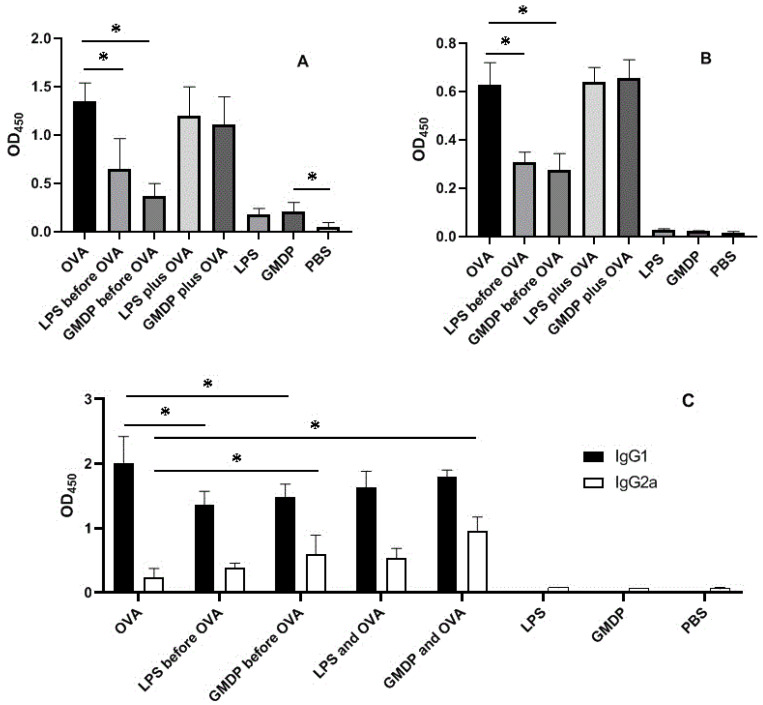
The amount of immunoglobulins in the bronchoalveolar lavage fluid and blood serum. (**A**) IgA content (1:20) in BALF; (**B**) the content of IgE (1:20) in the blood serum; (**C**) the content of IgG1 and IgG2a (1:1000) in the blood serum; * *p* < 0.05.

The study of the immunoglobulin status shows that in asthma, the amount of IgA in the BALF is significantly increased (Figure 3).

Normally, animals in BALF show trace amounts of IgA (group 8); GMDP and LPS slightly increase it (groups 6 and 7). At the same time, injections of GMDP and LPS prior to sensitization contribute to a significant decrease in IgA (2.6 times and 2.1 times, respectively) in BALF and IgE (2.2 times and 2.0 times, respectively) in the blood serum.

It is noteworthy that the effect of low-molecular-weight bioregulators of bacterial origin is different: lipopolysaccharide suppresses eosinophilia more strongly than GMDP, and GMDP is more effective than LPS in reducing the IgE class immunoglobulin although these differences are not statistically significant.

It should be noted that in the case of repeated administration of low-molecular weight-bioregulators of bacterial origin—lipopolysaccharide and GMDP before the introduction of the allergen—there is a slight decrease in the production of immunoglobulins of the IgG1 class and a significant increase in IgG2a, the most abundant immunoglobulin in mice, which performs a major role in the neutralization of foreign antigens.

## 4. Discussion

In the investigated mouse model of asthma, a double effect of LPS and GMDP was established: multiple administration of LPS and GMDP before sensitization reduces neutrophilia and eosinophilia and the levels of IgA in BALF and IgE in blood serum, while their joint administration together with an allergen significantly increases neutrophilia, eosinophilia, as well as IgA and IgE levels.

Our studies are supported by the results of Chinese scientists, who showed that LPS exposure 14 days prior to asthma can prevent future symptoms of OVA-induced murine asthma, with not only the pre-asthma interval being critical but also the amount of LPS administered [34]. The amount of LPS is critical in aggravating the severity of asthma, as it increases antigen-specific allergic reactions in the airways and inflammatory manifestations in patients and in experimental animal models with allergic asthma [35,36,37,38]. According to numerous epidemiological studies, it is the effect of low doses of LPS and bacterial components that is associated with protection against asthma in children raised in rural areas [3,39,40,41,42,43]. There are several theories that explain the established pattern. Proponents of the hygiene hypothesis argued that childhood infections and exposure to microbial components contribute to protection against allergic diseases, including asthma [44]. Since its inception, the hygiene hypothesis has been significantly revised, and the microbiome theory has been formed and revealed the importance of the endogenous microbiota inhabiting all mucous membranes, including human skin, for maintaining immune homeostasis as well as protecting against allergic diseases [45,46]. In this case, of course, it is necessary to take into account both genetic predisposition and violations of the barrier functions of the epithelium under the influence of environmental factors [47].

To explain the mechanisms of the results obtained in this study, it is necessary to consider changes at the molecular level, at the cellular level, and at the level of the microbiological community.

When considering the effect of LPS and GMDP on the induction of intracellular processes, a common feature is their interaction with a specific innate immunity receptor, activation of the transcription factor NF-κB, and induction of pro-inflammatory cytokines iNOS, IL-1, IL-6, and TNF-alfa and, as a result, triggering the inflammatory process. However, NF-κB, in addition to pro-inflammatory cytokines also triggers the synthesis of its own inhibitors, including IκBα and A20, forming negative feedback [48,49]. Moreover, negative feedback is delayed in time and develops much later than the actual inflammatory response. It is possible that it is the triggering of NF-κB inhibitors that underlies the effect of reducing the severity of the inflammatory process in the present study of experimental asthma, in a model of septic shock, as well as in some forms of psoriasis, when muramylpeptides taken during the remission period contributed to a decrease in the severity of the inflammatory process [50,51]. Despite the common ability for LPS and GMDP to activate NF-κB, the non-canonical pathways for them are different, and therefore, the final effects induced by LPS and GMDP may differ significantly. It should be kept in mind that the signal strength of NF-κB activation or inhibition depends on the initial state of the cell, accompanying stimuli, duration of exposure to agonists and their concentration, and many other parameters. Given the need to quickly relieve inflammation, which occurs particularly in surgery, various NF-κB inhibitors are being developed [52].

There are other explanations for the mechanism of reducing the intensity of the inflammatory process with repeated exposure to low-molecular-weight bioregulators of bacterial origin prior to sensitization and allergen provocation. One of them is the endosomal cleavage of the LPS complex with TLR4 and CD14, and the other is the ubiquitinated destruction of the GMDP complex with the NOD2 receptor. With the degradation of sensors, the ligands lose the ability to initiate an activating stimulus, and the intensity of inflammation will depend on the rate of biosynthesis of new receptors [53,54].

Thus, the decrease in the intensity of inflammation after multiple preliminary administration of low doses of GMDP and LPS can be explained by at least three mechanisms: (1) negative feedback, when the activating signal induces the appearance of its own inhibitors (for example, A20, IκBα, etc.); (2) endosomal degradation of the LPS complex with TLR4; and (3) ubiquitinated degradation of the NOD2 complex with GMDP. At the same time, taking into account the complexity, dynamics, and mutual influence of numerous intracellular processes, it can be assumed that reality is not limited to the listed mechanisms.

The decrease in the number of neutrophils in the lungs with prolonged exposure to GMDP can be explained by GMDP on the expression of surface markers in the presence of comitogenic factors. It was found that, under the influence of the bacterial peptide N-formyl-methionyl-leucyl-phenylalanine (fMLP) and GMDP on neutrophilic granulocytes in vitro, the effects of fMLP were neutralized: the number of neutrophils, including CD64+ and CD32+ decreased, and the expression density of CD11b also decreased [55]. It is also known that GMDP in vivo modulates the expression of surface molecules on functionally significant populations of dendritic cells (DC). In relation to the initial values in humans after taking the drug based on GMDP, the level of activated myeloid (MDC) and plasmacytoid (MPC) increased 1.9 times, while the ratio between these populations remained at the same level [56]. Moreover, the changes were related not only to quantitative but also to qualitative indicators: there was a statistically significant increase in the receptors of the chemokine CCR7, which is responsible for the rectification of DCs into the secondary lymphoid organs.

A similar decrease in IgE with the advance administration of LPS and GMDP prior to antigen sensitization explains the positive effect of the treatment of asthma and atopic diseases using GMDP, obtained in clinical practice [57,58,59]. Asthma is known to be caused primarily by the binding of complexes of the allergen and allergen-specific IgE to their receptors on antigen-presenting cells and the presentation of the processed allergen to T cells. These phenomena lead to mucus production, runny nose, itchy eyes, sneezing, airway hypersensitivity, and nasal congestion [60]. A decrease in IgE secretion is considered as the main etiological factor that reduces allergic inflammation and improves the course of the disease. While IgE is involved at an early stage of the inflammatory cascade and can be considered as the cause of allergic asthma, eosinophilia is considered to be a consequence of the entire process [61].

A decrease in immunoglobulin IgE is observed during drug therapy based on GMDP in children and adults with asthma and allergic diseases, provided that therapy is carried out during remission. At the same time, the ability of GMDP was found not only to reduce the severity of an allergic disease but also to reduce the number of seasonal diseases subject to several courses. One course includes taking the drug Licopid 1 mg based on GMDP for 10 days, then a 20-day break. It is assumed that one of the manifestations of the positive effect of GMDP in asthma may be a shift in the Th1/Th2 balance towards Th1 [62]. In addition, the discovered ability of GMDP to stimulate the production of interferon-gamma can serve as an explanation for the preventive effect in viral diseases [56].

Furthermore, the ability of GMDP revealed in this investigation to increase the IgG titer when administered together with the antigen correlates with the known data on the adjuvant properties of muramylpeptides [63,64].

When translating the results of a study in an experimental model of asthma on the effectiveness of low-molecular-weight bioregulators in humans, it is necessary to take into account that GMDP, as in the case of LPS, showed a positive effect in the asthma model, being injected multiple times at low concentrations before the allergen was administered to healthy mice, and as shown in this study, when administered to mice with asthma, the severity of allergic manifestations was aggravated. In addition, it should be taken into account that the classic OVA-induced mouse model of asthma with Alum belongs to the Th2-high endotype, while in humans, along with Th2-high, non-Th2-high and Th2-low endotypes are present [65].

The results of this study can serve as confirmation that insufficient exposure to antigens of a bacterial nature, in particular, with prolonged use of antibiotics, creates the prerequisites for excessive reactivity to harmless antigens and can be compensated for by means based on low-molecular-weight bioregulators of bacterial origin, which replenish the missing signals for receptors of innate immunity, the constant activation of which at some level is necessary to maintain homeostasis.

The results obtained provide a new understanding of the clinical course of asthma and can be used to optimize the regimens for withdrawal from biologics, which are already possible in a certain category of patients [66]. At the same time, low-molecular-weight bioregulators can have an effect both on predictors of the early course of asthma and on markers of the late stages of allergic inflammation during remission, but their use in the acute phase of allergic inflammation will aggravate the severity of asthma.

## 5. Conclusions

The study confirmed the key importance of innate immunity in asthma. Normally, bacterial components contribute to the classic protection of the lungs: in bronchoalveolar lavage, under the influence of GMDP and LPS, the level of macrophages, lymphocytes, and IgA immunoglobulins increases. In asthma, the effect of GMDP and LPS is multidirectional: when they are repeatedly administered before sensitization, the bacterial components noticeably reduce the severity of the allergic process.

In the developed experimental models of asthma, the ability of LPS and GMDP to influence both cellular and humoral immunity has been established; the key importance of repeated preliminary exposure to low-molecular-weight bioregulators of bacterial origin in protection against asthma has been demonstrated.

## Figures and Tables

**Table 1:** Experimental design and protocol.

Day	Animal Groups							
	1 Asthma (OVA)	2 LPS before OVA	3 GMDP before OVA	4 LPS and OVA	5 GMDP and OVA	6 LPS	7 GMDP	8 Phosphate Buffer (PBS)
−5		LPS i/p	GMDP i/p			LPS i/p	GMDP i/p	PBS i/p
−4		LPS i/p	GMDP i/p			LPS i/p	GMDP i/p	PBS i/p
−3		LPS i/p	GMDP i/p			LPS i/p	GMDP i/p	PBS i/p
−2		LPS i/p	GMDP i/p			LPS i/p	GMDP i/p	PBS i/p
−1		LPS i/p	GMDP i/p			LPS i/p	GMDP i/p	PBS i/p
0	OVA i/p			OVA+LPS i/p	OVA+GMDP i/p			PBS i/p
1								
…								
13								
14	OVA i/p			OVA+LPS i/p	OVA+LPS i/p			PBS i/p
15								
16								
17								
18								
19								
20								
21	OVA i/p			OVA+LPS i/p	OVA+LPS i/p			PBS i/p
22								
23								
24								
25								
26								
27	OVA i/n					PBS i/n	PBS i/n	PBS i/n
28	OVA i/n					PBS i/n	PBS i/n	PBS i/n
29								
30								
31								
32								
33								
34								
35	OVA i/n					PBS i/n	PBS i/n	PBS i/n
36								
37	BAL, lung and sera collecting							

Note: OVA, ovalbumin; GMDP, glucosaminylmuramidipeptide; LPS, lipopolysaccharide; PBS, phosphate buffered saline; i/p, intraperitoneal administration; i/n, intranasal administration; BAL, bronchoalveolar lavage.

## Data Availability

Not applicable.
